# Developmental transcriptomes predict adult social behaviours in the socially flexible sweat bee, *Lasioglossum baleicum*

**DOI:** 10.1111/mec.17244

**Published:** 2023-12-18

**Authors:** Kennedy S. Omufwoko, Adam L. Cronin, Thi Thu Ha Nguyen, Andrew E. Webb, Ian M. Traniello, Sarah D. Kocher

**Affiliations:** ^1^ Department of Ecology and Evolutionary Biology Princeton University Princeton New Jersey USA; ^2^ Lewis‐Sigler Institute for Integrative Genomics Princeton University Princeton New Jersey USA; ^3^ Department of Biological Sciences Tokyo Metropolitan University Tokyo Japan; ^4^ Howard Hughes Medical Institute Princeton University Princeton New Jersey USA

**Keywords:** evolution, genomics, plasticity, social behaviour

## Abstract

Natural variation can provide important insights into the genetic and environmental factors that shape social behaviour and its evolution. The sweat bee, *Lasioglossum baleicum*, is a socially flexible bee capable of producing both solitary and eusocial nests. We demonstrate that within a single nesting aggregation, soil temperatures are a strong predictor of the social structure of nests. Sites with warmer temperatures in the spring have a higher frequency of social nests than cooler sites, perhaps because warmer temperatures provide a longer reproductive window for those nests. To identify the molecular correlates of this behavioural variation, we generated a de novo genome assembly for *L. baleicum*, and we used transcriptomic profiling to compare adults and developing offspring from eusocial and solitary nests. We find that adult, reproductive females have similar expression profiles regardless of social structure in the nest, but that there are strong differences between reproductive females and workers from social nests. We also find substantial differences in the transcriptomic profiles of stage‐matched pupae from warmer, social‐biased sites compared to cooler, solitary‐biased sites. These transcriptional differences are strongly predictive of adult reproductive state, suggesting that the developmental environment may set the stage for adult behaviours in *L. baleicum*. Together, our results help to characterize the molecular mechanisms shaping variation in social behaviour and highlight a potential role of environmental tuning during development as a factor shaping adult behaviour and physiology in this socially flexible bee.

## 1 INTRODUCTION

**FIGURE 1:**
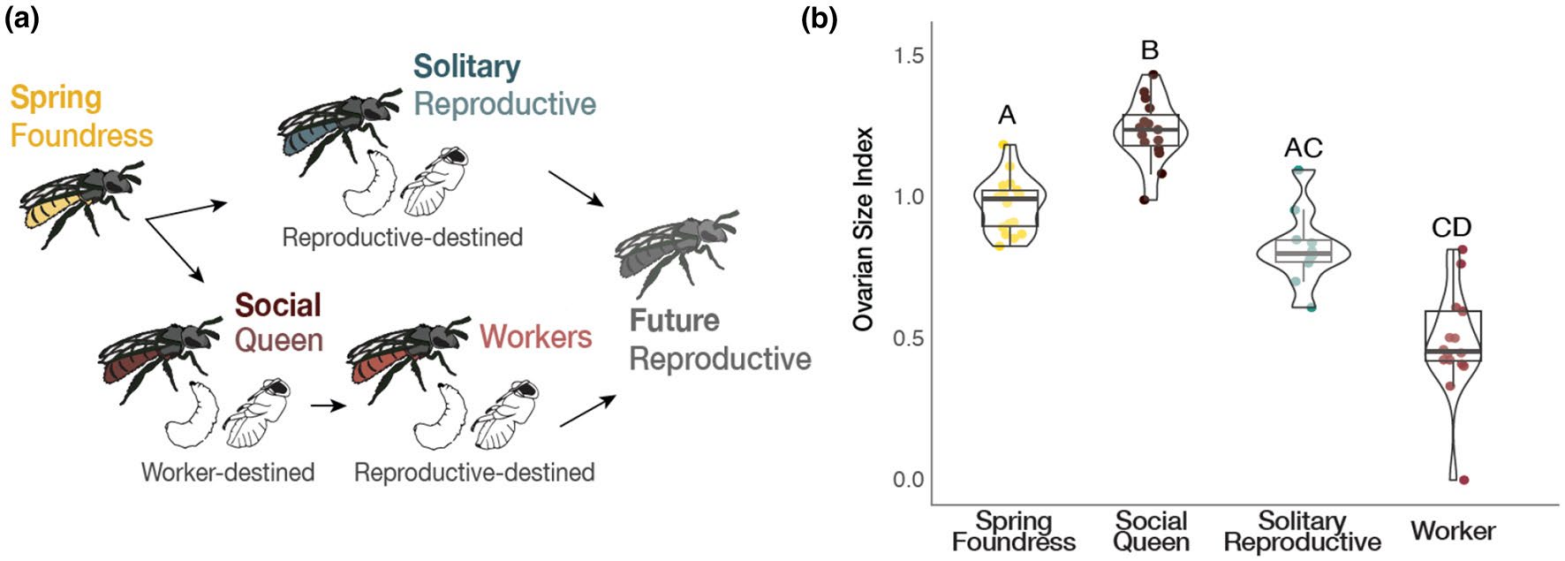
*Lasioglossum baleicum* is a socially flexible sweat bee. (a) In the spring, individual females (spring foundresses) can either reproduce solitarily or form eusocial nests with workers in the summer (Cronin & Hirata, 2003). This variation is dependent on environmental conditions – females tend to produce eusocial nests in warmer patches and solitary nests in cooler patches of an aggregation near Sapporo in Hokkaido, Japan. (b) Comparisons of the ovarian size indices (length of the longest oocyte [mm]/intertegular distance [mm]) reveal that social queens (*n* = 15) have the greatest ovarian activation (Kruskal–Wallis: $\chi_{3}^{2}$ = 51.78, *p* = 3.34e‐11). Solitary reproductives (*n* = 11) are intermediate, and workers (*n* = 17) had the least developed ovaries. Letters represent significant differences according to Dunn's post‐hoc tests.

Systems that encompass natural variation in behaviour can help to unmask the genetic and environmental factors that shape these complex traits. The halictine bee, *Lasioglossum baleicum*, is a socially flexible bee species capable of producing both eusocial and solitary nests (Figure 1). This sweat bee is common in the temperate regions of Japan, where nests at lower elevations and latitudes are primarily eusocial, while high‐elevation populations produce all solitary nests (Hirata & Higashi, 2008). Beyond this clinal variation in eusociality, some populations in Northern Japan include a mix of eusocial and solitary nests at the same site, with social nests found primarily in sunny patches of the nesting aggregation and solitary nests found primarily in the shade (Cronin & Hirata, 2003; Hirata & Higashi, 2008; Yagi & Hasegawa, 2011). Thus, *L. baleicum* females can produce both eusocial and solitary nests, and this variation can occur both across different populations and within a single population.

The behavioural variation encompassed by *L. baleicum* is likely to be adaptive. Eusocial nests have almost a ninefold increase in reproductive success compared to solitary nests, and the inclusive fitness benefits to workers have been calculated to be higher than those of their solitary counterparts (Yagi & Hasegawa, 2012). Despite these reproductive benefits, eusociality may not always be the most successful strategy. Solitary nests are more common than eusocial ones both at higher elevations and in cooler, shady sites (Hirata & Higashi, 2008). The cooler temperatures in these regions are likely to result in shorter seasonal windows where temperatures are warm enough for bees to forage and for brood to develop but limit reproduction to one brood per season (Kocher et al., 2014). While solitary females produce the next generation of reproductives, the eusocial strategy requires an initial investment in worker production followed by the production of a second, reproductive brood. Thus, in cooler areas, the developmental windows may not be sufficiently long for eusocial nests to complete their reproductive cycles (Kocher et al., 2014). As a result, there appears to be a trade‐off between the fitness benefits associated with a eusocial reproductive strategy that initially invests in the production of workers versus the amount of time required for a nest to produce the next generation of reproductive females. This trade‐off and the importance of season length as a driver of social evolution has been described and documented more broadly across many insects (Cronin & Schwarz, 1999a; Davison & Field, 2016, 2018; Field et al., 2012; Kocher et al., 2014; Plateaux‐Quénu et al., 2000; Schwarz et al., 2007; Sheehan et al., 2015).

Whether an adult female remains in a nest as a worker or disperses to become a solitary reproductive is largely flexible in adulthood (Breed et al., 1978; Brothers & Michener, 1974; Steitz & Ayasse, 2020). In *L. baleicum* and all social halictines and paper wasps, females are capable of reproduction throughout their lives (i.e., they are totipotent; Hirata & Higashi, 2008). In the absence of a queen, workers can dynamically activate their ovaries and take over as a new dominant, reproductive female in the nest. As a result, most work in sweat bees has focused on the behavioural interactions and chemical signals experienced by adults within the nest. However, developmental factors are also likely to influence adult behaviour and physiology, including differences in nutrition (Kapheim et al., 2011; Lawson et al., 2017) and temperature (DeHaan et al., 2022; Hirata & Higashi, 2008). Reproductive females typically eclose with greater fat stores (Brand & Chapuisat, 2012), and, in many lineages, these females are often larger than workers. Thus, the social flexibility observed in *L. baleicum* is likely to result from a complex interplay of developmental and social environments.

To better understand the factors that shape and potentially maintain social flexibility in *L. baleicum*, we characterized the environmental and molecular correlates of the different behavioural strategies found within a single population of this species located near Sapporo, Japan. In this population, females either produce a solitary nest with a single, mixed‐sex brood or eusocial nests with an initial female‐biased worker brood followed by a mixed‐sex reproductive brood, typically separated by an inactive period of several weeks (Figure 1a; Cronin & Hirata, 2003; Davison & Field, 2018). We first examined the relationship between soil temperature and nest social structure throughout the active season. Then, we characterized differences in gene expression patterns across key developmental timepoints and social phenotypes. To this end, our study highlights the interplay between environmental variation and individual physiology in modulating social behaviour in this unique, socially flexible bee.

## 2 MATERIALS AND METHODS

### 2.1 Study organism and sample collections

*L. baleicum* individuals were collected from Nishioka Park in Japan (near Sapporo, western Hokkaido; alt. 150 m, 141°35′ E, 43°00′ N). Temperature probes (IBS‐TH2, INKBIRD) were placed in the soil at approximately 20 cm depth to approximate the position of the brood clusters. Data were collected during two trips: the first in June 2021 and the second in July 2021.

Nests in this aggregation contain a mix of solitary and eusocial nesting strategies (Figure 1a). Solitary nests consist of a single, reproductive female solely responsible for provisioning her brood. This brood is a mix of reproductive males and females produced in a single bout of foraging and egg laying. Eusocial nests consist of multiple females, with a single queen and an average of 2–3 workers provisioning the brood. Social queens produce two broods: first a female‐biased brood of workers followed by a mix of reproductive males and females (gynes). Like many other halictine bees, males disperse after eclosion. All females are morphologically similar, and eusocial workers are capable of mating and reproduction in the absence of a queen. Many mid‐season nests contain multiple adult females, with some evidence of nest swapping among workers (Yagi & Hasegawa, 2012).

**FIGURE 2:**
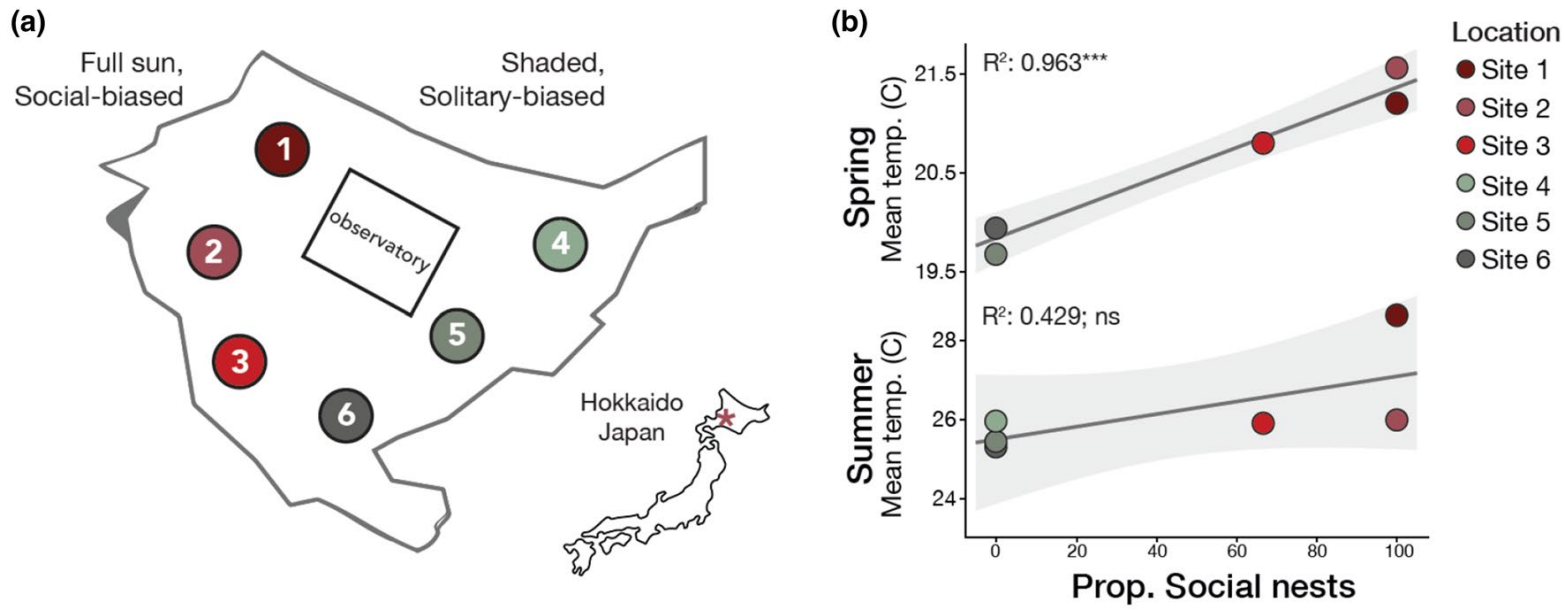
Spring soil temperatures predict nesting behaviour. (a) Nests were studied at six locations throughout an aggregation in Hokkaido (*n* = 19, 3, 4, 7, 9, 6 nests excavated at each site, respectively). The aggregation is found in an open lawn surrounded by trees. Sites 1–3 receive full sun, and sites 4–6 experience higher shade. (b) Temperature probes placed in the soil during spring collections were strongly correlated with nesting behaviour (shown here as the proportion of social nests at a given site) in the spring (linear regression, *R* ^2^ = .963, *p* = 5.08e‐4) but not in the summer (linear regression, *R* ^2^ = .429, *p* = .158). Lines indicate the linear fit of the regressions and shading indicates the 95% confidence intervals for the model.

Samples were collected at two different timepoints. The first collection (spring) was done on June 16, 2021, when nests contained spring foundresses, pupae from brood one, and no adult offspring. The second collection (summer) was July 28, 2021, when nests contained founding females (solitary reproductives or queens), adult workers (social nests only), and developing broods (eggs, larvae, pupae). Collections began at 04:00 by blocking the nest entrance with a plastic cup to ensure none of the adults escaped when foraging time approached. During excavation, white talcum powder was injected into each individual nest entrance with a Pasteur pipette to distinguish individual nest tunnels and architecture, facilitating collection of individuals belonging to the focal nest. To assess environmental correlates, temperature probes were placed into the soil in a central location relative to several nests. These probes were inserted at six different sites (three sunny sites and three shady sites) on the first day of nest excavations and remained in the soil for 72 h at each location. Excavated nests were proximal to these six probes (Figure 2a). This procedure was followed for both the spring and summer collecting trips. Nests were classified as social nests when at least three individuals were found in the same nest. On site, a combination of factors was used to identify workers, including the presence of pollen on abdomens, wing wear, smaller body sizes, and proximity to nest entrance. Queens were typically the last individuals found during excavations and were located at the bottom of the nest. These behavioural classifications were later confirmed using ovary dissections; queens had fully developed ovaries with mature eggs present while workers had lower or no ovarian activation. We selected nests to excavate that were proximal to our temperature probes, and this information was recorded in the sample metadata. To assess relationships between soil temperature and sociality, we calculated the proportion of social nests as the number of social nests at a given site divided by the total number of nests sampled at that site. Individual bees were placed in labelled 1.5 mL cryoresistant microcentrifuge tubes and immediately flash frozen in liquid nitrogen in the field.

We collected a total of 62 adults and 22 pupae for this study from 34 nests: eusocial queens (*n* = 15), spring foundresses (*n* = 19), solitary reproductives (*n* = 11), eusocial workers (*n* = 17), spring worker‐biased female pupae (*n* = 11), and spring solitary‐biased female pupae (*n* = 11; Table S1). For classifying spring pupae as solitary‐ or worker‐biased, we relied on the nesting location at the site. Because the entire nests were excavated and pupae were flash frozen in the spring, we could not test their individual behaviours as adults. However, spring nests on the sunny side showed a strong female‐biased sex ratio among pupae consistent with a eusocial nesting strategy. Female pupae excavated from nests proximal to the sunny side temperature probes in the spring were considered “worker‐biased” pupae, while spring pupae collected proximal to the shady‐side temperature probes were classified as “solitary‐biased”. We collected both male and female pupae and flash froze them in the field, but only female pupae were used for downstream studies. Female pupae used for gene expression analyses were stage‐matched based on cuticle and eye pigmentation; we selected late‐stage pupae with no pigmentation on cuticles and light pink eyes (Tian & Hines, 2018). It takes about 2.5 weeks for eggs to eclose and transition to late‐stage pupae with no cuticle pigmentation and light pink eyes. We did not include males in this study as they do not contribute to nest activities and disperse soon after they emerge.

### 2.2 Ovary dissections, phenotypic measurements, and caste assignments

Abdomens were dissected in RNAlater ICE under a Leica M125 microscope, and pictures of ovaries were captured with the Leica MC190 HD camera. We measured intertegular distance (IT span) as a proxy for body size and measured the length of the longest oocyte to quantify ovarian development. These measures were taken for all adult bees used for gene expression analysis in order to infer caste differences among the adults (as in Kapheim et al., 2012). Data are included in Table S1.

To control for differences in body size, we calculated the ovarian index for each bee by taking the measurement of the longest oocyte and dividing it by IT span (Saleh & Ramírez, 2019). We measured both ovary and oocyte lengths since they can differ depending on the reproductive maturity. All individuals were measured blindly to prevent any bias in body size measurements. Ovary and oocyte lengths were quantified using ImageJ v1.53n. Because ovary length distributions deviated from normality, we implemented Kruskal–Wallis tests followed by post‐hoc Dunn's pairwise tests to assess relationships between body and ovary size differences across behavioural groups. All analyses were carried out in R (R Core Team, version 4.1.2); *p*‐values were adjusted for multiple comparisons using the Benjamini–Hochberg method.

### 2.3 Genome sequencing and assembly

A de novo genome for *L. baleicum* was generated using 10x genomics linked‐reads technology. The assembly was generated from a netted, foraging female (of unknown nest ID) from the same site our collections were conducted. We did not dissect ovaries to determine reproductive status because it would not impact genomic DNA, and there is no evidence for genetic differences between social and solitary forms of bees in this aggregation (Hirata & Higashi, 2008). DNA was extracted from a single, frozen individual collected from Nishioka Park using a Qiagen Genomic Tip kit (Qiagen, USA, Catalogue #10223) to isolate high‐molecular‐weight DNA. Libraries were sequenced to approximately 40x coverage. An assembly with a scaffold N50 of 1.9 Mb was generated using Supernova v2.1.1 (Weisenfeld et al., 2017), and subsequent filtering and other quality control methods followed those of (Jones et al., 2023). The assembled genome is available on GenBank, accession GCA_022376115.1. Genome assembly statistics and completeness are presented in Table S2 (compleasm/miniBUSCO; Huang & Li, 2023).

### 2.4 Gene annotations

Gene annotations were initially predicted by BRAKER v2.1.6 (Brůna et al., 2021) using a two‐run procedure. The first run derived predictions from protein hints using the *L. baleicum* repeat‐soft‐masked assembly. To soft‐mask our assembly, we began by using RepeatModeler v1.0.8 to annotate repeats in our assembly (Smit et al., 2008). The annotations were then clustered using cd‐hit‐est v4.6 and filtered to remove any putative elements that were found to be homologous to proteins in either Uniprot or FlyBase using BLASTX v2.5.0+ (Camacho et al., 2009; Li & Godzik, 2006). The filtered annotations were then used as the library for RepeatMasker v4.1.0 to produce the soft‐masked assembly (Smit et al., 2008). Protein hints were generated by mapping a collection of orthologous Arthropoda proteins downloaded from OrthoDB v10 (Kriventseva et al., 2019) to the *L. baleicum* assembly using ProtHint v2.6.0 (Tom et al., 2020). OrthoDB was recently updated to v11, but the v10 database can be found here https://v100.orthodb.org/. The Arthropoda data used in this analysis can be accessed at this link: https://v100.orthodb.org/download/odb10_arthropoda_fasta.tar.gz. The homology‐based predictions resulted in 19,723 genes from BRAKER, 19,723 genes from AUGUSTUS alone, and 16,950 genes from AUGUSTUS ab initio.

The second run combined RNAseq libraries from the brains of three queens, three workers, three solitary reproductives, and three spring foundresses. To generate predictions with UTRs, reads were aligned using STAR v2.7.5c (Dobin et al., 2013). These predictions resulted in 42,845 genes from BRAKER, 42,846 genes from AUGUSTUS alone, and 10,665 genes from AUGUSTUS ab initio.

The results of the two‐run predictions were then combined using TSEBRA v1.0.2 (Gabriel et al., 2021) and fed into MAKER v3.01.04 (Cantarel et al., 2008) as ab initio predictions. Finally, we also provided MAKER with protein sequences from *Bombus impatiens* (GCF_000188095.3_BIMP_2.2, UP000515180) and *Apis mellifera* (Amel_HAv3.1, UP000005203) to help locate coding regions and gene boundaries (Campbell et al., 2014). To better recognize intron/exon boundaries, infer alternate splice variants, and identify UTRs, we provided EST evidence from reconstructed transcripts generated by PASA v2.5.0 (Haas et al., 2003) from RNAseq reads assembled by Trinity v2.13.2 (de novo assembly and genome‐guided; Grabherr et al., 2011). We also included the always_complete option to require gene models to contain both start and stop codons and the correct_est_fusion option to avoid merging of gene models due to overlapping UTRs. The output of MAKER includes both predictions that overlap the provided evidence (e.g., protein homology, EST evidence, etc.) and non‐overlapping predictions. To confirm if predictions without evidence may represent protein‐coding genes missing from our annotations, we used InterProScan v5.52‐86.0 (Jones et al., 2014) to identify those predictions with evidence of InterPro families; gene predictions with InterPro matches were included, while those that did not have an InterPro match were discarded. Finally, we reran MAKER with the original evidence‐overlapping annotations, and we included the non‐overlapping predictions with evidence of InterPro families as ab initio predictions; the resulting annotations were then used in this analysis. BUSCO analyses (Simão et al., 2015) were conducted to assess the completeness of gene annotations (Table S2).

### 2.5 Tissue dissections and RNA extractions

Heads were immersed in a dry ice/ethanol bath, and the frons and mandibular regions were removed to expose the brain. Then, tissues were submerged in either 200 μL (heads) or 400 μL (abdomens) of RNAlater ICE (Invitrogen AM7030) and stored overnight at −20°C. The following day, all heads and abdomens were dissected in RNAlater ICE, and the brains and fat bodies were stored at −80°C until further processing. For RNA extraction, tissues were homogenized with a Qiagen TissueLyser, and mRNA was extracted using magnetic mRNA isolation with Dynabeads (Invitrogen, 61011) according to the manufacturer's protocol. Cleaned mRNA was quantified with a Qubit high sensitivity (HS) assay kit (Invitrogen, Q32852), and the integrity of RNA was estimated by electrophoresis using TapeStation 2200 with High Sensitivity D5000 ScreenTape (Agilent Technologies).

### 2.6 cDNA library preparation and sequencing

Individual libraries were constructed using NEBNext Ultra II Directional Prep Kit for Illumina (NEB #7760, New England Biosystems, Ipswich, Massachusetts) and sequenced at the Princeton Genomics Core. A MiSeq run was conducted for initial library quantification and quality controls. Libraries were then rebalanced and sequenced on an Illumina NovaSeq 6000 using v1.5 reagent kits with paired, 150 bp reads and approximately 10 million reads per library. RNA extractions, library preparations, and sequencing were randomized with respect to tissues and sex to avoid batch effects. One sample was excluded from downstream analyses due to library preparation failure. Raw transcriptomic reads are available from NCBI SRA archive under bioproject ID PRJNA884745. Reads were combined for downstream analyses.

### 2.7 Quality filtering, read mapping, and quantification

The resulting fastq files were demultiplexed using deML, a maximum likelihood demultiplexing algorithm that allows probabilistic sample assignments, with default parameters and accounting for one set of reverse‐complement indices (Renaud et al., 2015). Filtering was performed to remove any remaining adapter sequences using fastp (Chen et al., 2018). Illumina strand‐specific paired‐end sequences from the NovaSeq reads were quality‐filtered using fastp with a 4‐bp window size, and a quality threshold of 20 with an option to detect and remove adaptors for paired‐end sequences. All reads below 50 bp were discarded. Read qualities were calculated with FastQC (bioinformatics.babraham.ac.uk/486 projects/fastqc/). We aligned the filtered FASTQ sequences to their respective reference genome, *L. baleicum*, with STAR v2.7.5c (Dobin et al., 2013). Subsequent fragment counts for our annotated genes were then generated using featureCounts v2.0.1 (Liao et al., 2014). We note that our read counts assigned to genes were lower than the recommended 10 million reads per sample threshold (Conesa et al., 2016); this was due to a large proportion of ribosomal RNA in the resulting sequences and likely decreased our ability to detect differentially expressed genes at lower expression levels. However, this should not substantially influence our ability to detect global differences in gene expression among different groups. Gene expression levels were quantified using exon annotations and summarized at the gene level (Table S3).

### 2.8 Differential gene expression analysis

#### 2.8.1 Global expression patterns and normalization

Analyses of differential gene expression among behavioural groups were carried out with the DESeq2 package in R (Love et al., 2014). To account for hidden batch effects, we used surrogate variable analysis (SVA) to identify and estimate the unknown variations (Leek & Storey, 2007). Samples with low coverage (<2 million reads) were excluded from downstream analyses, and five brain tissues and eight fat body tissues were filtered out using these criteria. All the brain tissues of spring pupae passed the threshold except one pupal fat body sample, which was subsequently excluded. To avoid problems of estimating dispersion due to low expressed features, we filtered out rows that had normalized gene counts of less than 2 in at least 10 samples. This was done independently for both the brain and fat body datasets. After filtering, there were 11,854 adult brain genes, 10,665 adult fat body genes, 11,248 pupal brain genes, and 10,342 pupal fat body genes that remained for analysis. After filtering, the following sample sizes remained for the brain transcriptomes: workers (*n* = 16), queens (*n* = 14), solitary reproductives (*n* = 10), solitary‐biased pupae (*n* = 11), and social‐biased pupae (*n* = 11). After filtering, the following sample sizes remained for the fat body transcriptomes: workers (*n* = 16), queens (*n* = 14), solitary reproductives (*n* = 9), solitary‐biased pupae (*n* = 8), and social‐biased pupae (*n* = 11). Differentially expressed genes (DEGs) were called in DESeq2 using a contrast approach to test for behavioural effects on gene expression in all the genes that passed the filtering threshold in both brains and fat bodies. To correct for multiple testing, we employed a Benjamini–Hochberg adjustment (Benjamini & Hochberg, 1995). Genes with a false discovery rate (FDR) < 0.001 were considered differentially expressed.

A principal component analysis was used to visualize global patterns in gene expression among samples and tissues. Expression heat maps were constructed using the “heatmaps” package in R (Perry, 2022). Overlaps of DEGs were inspected using intersection plots that were generated by UpsetR package 1.4.0 (Conway et al., 2017). Volcano plots were produced with the “Enhanced volcano” R package (Kevin blighe, 2022). Normalized counts were used to generate hierarchical heatmaps for brains and fat bodies using the “heatmap” function in R.

#### 2.8.2 Gene ontology enrichment analyses

To identify Gene Ontology (GO) terms overrepresented within our DEGs, we first used Trininotate (Bryant et al., 2017) to assign GO terms to *L. baleicum* genes. We then performed GO analysis on DEGs from each behavioural or developmental contrast in the brain and in the fat body (separately). We used the “weight01” algorithm with Fisher's exact test in the R package, TopGO (Alexa & Rahnenführer, 2007). We analysed the top 20 terms in the Biological Process class, separating DEGs into upregulated and downregulated sets from each contrast: workers versus reproductive adult brains and the brains of worker‐biased pupae from full sun sites versus reproductive‐biased pupae from the shaded sites (see below for further details). A full set of contrasts and their GO enrichment results for all social forms and tissues can be found in Table S10.

### 2.9 Pupal brain DEG predictions of adult behavioural state

Using normalized, variance‐stabilized transformed counts of genes differentially expressed between reproductive‐ and worker‐biased pupal brains (“pupal DEGs”), we evaluated a random forest algorithm's classification accuracy of adult behavioural state when trained on either all 1121 pupal DEGs or random subsets of 10, 50, 100, or 500 pupal DEGs. Each classification for pupal DEGs was compared to a random, equivalently sized subset of non‐pupal DEGs (“Random genes”) or pupal DEGs with adult behavioural state label shuffled (“Shuffled labels”). Each analysis was iterated 1000 times using the randomForest package in R (Liaw & Wiener, 2002).

## 3 RESULTS

### 3.1 Body size does not vary among behavioural forms, but social queens have the largest ovaries

Intertegular distance, a proxy for body size, did not vary significantly among the queens, workers, foundresses, or solitary reproductive females (Figure S4; Kruskal–Wallis: $\chi_{3}^{2}$ = 3.47, *p* = .32). However, there were significant differences in ovarian development (measured as the ovarian index; the length of the longest oocyte/intertegular distance). Queens had the highest ovarian index compared to other groups, with solitary reproductives intermediate, and workers with the smallest index (Figure 1b; Kruskal–Wallis: $\chi_{3}^{2}$ = 51.78, *p =* 3.34e‐11). A complementary analysis considering solely the longest oocyte (not normalized to body size) produced similar results (Kruskal–Wallis: $\chi_{3}^{2}$ = 52.52, *p =* 2.32e‐11).

### 3.2 Nest social structure is strongly correlated with spring soil temperatures

*L. baleicum* nests in large aggregations with hundreds to thousands of nests present in a single aggregation. In these aggregations, the peripheral nesting sites are shadier, cooler, and more humid (Figure 2a), and nests in these locations tend to be solitary (containing a single reproductive female). Nests constructed in the warmer, sunnier regions of the aggregation tend to be eusocial (e.g., contain reproductive queens and non‐reproductive workers). Soil probes placed at each of the sites revealed that spring soil temperatures are tightly correlated with nest social structure (Figure 2b; linear regression, *R* ^2^ = .963, *p* = 5.08e‐4). However, summer soil temperatures did not show a significant correlation with social structure of nests (linear regression, *R* ^2^ = .429, *p* = .158). We henceforth refer to the warmer spring soil temperature sites (sites 1–3) as “social‐biased” sites and the cooler spring temperature sites (sites 4–6) as “solitary‐biased” sites.

### 3.3 Adult gene expression patterns distinguish reproductive state

**FIGURE 3:**
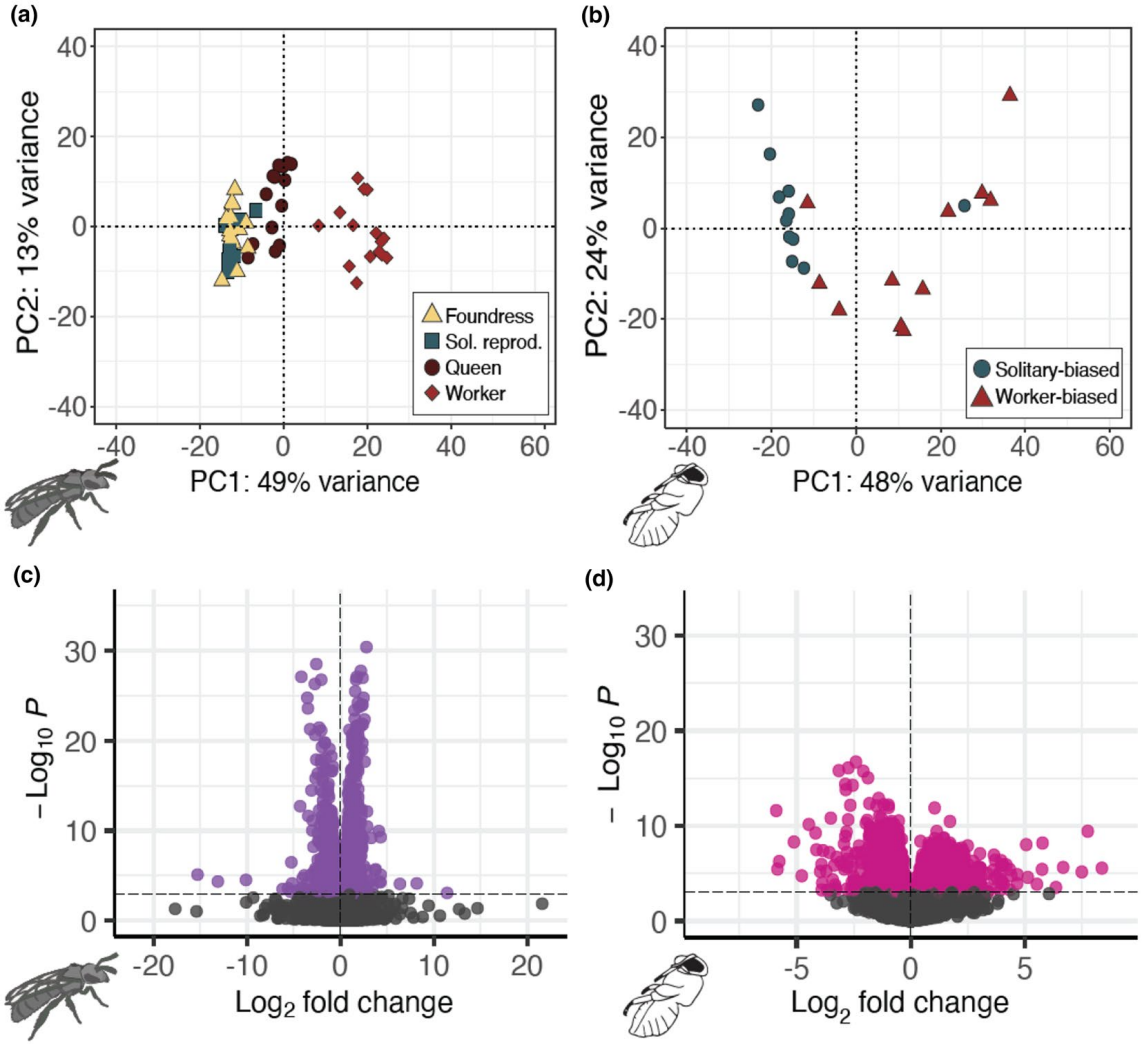
Adult and pupal brain transcriptomes reflect environmental conditions and social behaviour. (a) Adult workers (*n* = 16) have transcriptomic profiles highly distinguishable from reproductively active foundress (*n* = 14), queen (*n* = 14), and solitary reproductive (*n* = 10) females. PC1 shows a strong positive correlation with ovarian development (*F*_1,52_ = 32.55, *R* ^2^ = .37, *p* = 5.592e‐07). (b) PCA on all pupal brain transcripts discriminates between late spring pupae excavated from the social‐biased (*n* = 11) and the solitary‐biased (*n* = 11) sites. Pupae from social‐biased sites are most likely worker‐destined, while pupae excavated from solitary‐biased sites are predicted to become reproductive females. (c and d) Volcano plots highlighting DEGs of (c) workers vs. reproductive adults (queens and solitary reproductives), *n* = 1158 DEGs, and (d) Solitary‐biased vs. social‐biased pupae (*n* = 1121 DEGs). DEGs were determined with DEseq2 (FDR < 0.001).

We next compared the brain and fat body gene expression profiles of several behavioural phenotypes of adult females: spring foundresses, solitary reproductives, and queens and workers from social nests (Table S5; Figures S6–S8). A principal component analysis using all transcripts that passed our initial filters separates non‐reproductive workers from solitary reproductives, queens, and foundresses. Thus, the largest contribution to variation in gene expression appears to be reproductive state (Figure 3a). Similar patterns are also observed in the fat bodies (Figure S9).

To gain a better understanding of which genes distinguish workers from reproductive females, we next tested for genes differentially expressed between solitary reproductives and queens versus workers and identified 1585 differentially expressed genes in the brain (DEGs; FDR < 0.001; Figure 3b). GO enrichment analyses (Table S10) reveal several Biological Processes that are associated with gene expression in workers, including protein transport and long‐term memory (Figure S11a). Similarly, genes upregulated in the brains of reproductive females are enriched for transcription and translation (Figure S11b). Genes associated with transcription and translation are upregulated in the fat bodies of reproductives (Figure S12a), while genes associated with mitochondrial function and metabolism are upregulated in worker fat bodies (Figure S12b).

We chose not to include foundresses in these analyses because they were sampled at an earlier timepoint in the season, but we note that there were 482 brain DEGs and 294 fat body DEGs between all sets of reproductive females compared to workers (Figures S7 and S8). The results of all pairwise contrasts are summarized in Table S5.

### 3.4 Pupal gene expression patterns are correlated with spring soil temperatures and predict adult behaviour

Because spring soil temperatures were strongly correlated with the social structure of the nests (Figure 2), we were interested in comparing the gene expression profiles of stage‐matched developing pupae in the shady, solitary‐biased sites versus the sunny, social‐biased sites. We used female pupae collected from the first spring brood. Thus, pupae located in nests from solitary‐biased sites are likely to develop into solitary reproductive females, while pupae collected from social‐biased sites should be more likely to develop into workers.

We sequenced the brain and fat body transcriptomes, and used all of transcripts that passed our initial filters in a principal component analysis. The analysis separates pupal transcriptomes into two primary clusters along the first principal component (PC1, Figure 3b). PC1 explains 48% of the variance among samples and is strongly correlated with nesting locality (Figure 3b; Social‐biased vs. Solitary‐biased, *F*_1,20_ = 16.79, *R* ^2^ = .4292, *p* = .00056). The same analyses conducted on the pupal fat bodies also uncovers a similar association between PC1 and nesting locality (Figure S9; *F*_1,17_ = 27.47, *p* = 6.636e‐05, *R* ^2^ = .5952).

We then performed a differential expression analysis between these stage‐matched pupae from solitary‐biased versus social‐biased sites (Table S5; Figure S13). We identified 1121 genes differentially expressed in pupal brains (Figure 3d). Genes upregulated in social‐biased versus solitary‐biased pupa brains are enriched for synaptic transmission, and olfactory learning is also enriched but to a lesser degree. Genes downregulated in social‐biased versus solitary‐biased brains are enriched for cell division, and protein folding is also enriched to a lesser degree (Figure S14). Of these 1121 genes, 275 are also differentially expressed between adult reproductives and workers, and this overlap is more than expected by chance (hypergeometric test, *p* = 4.49e‐23; odds ratio = 2.19).

**FIGURE 4:**
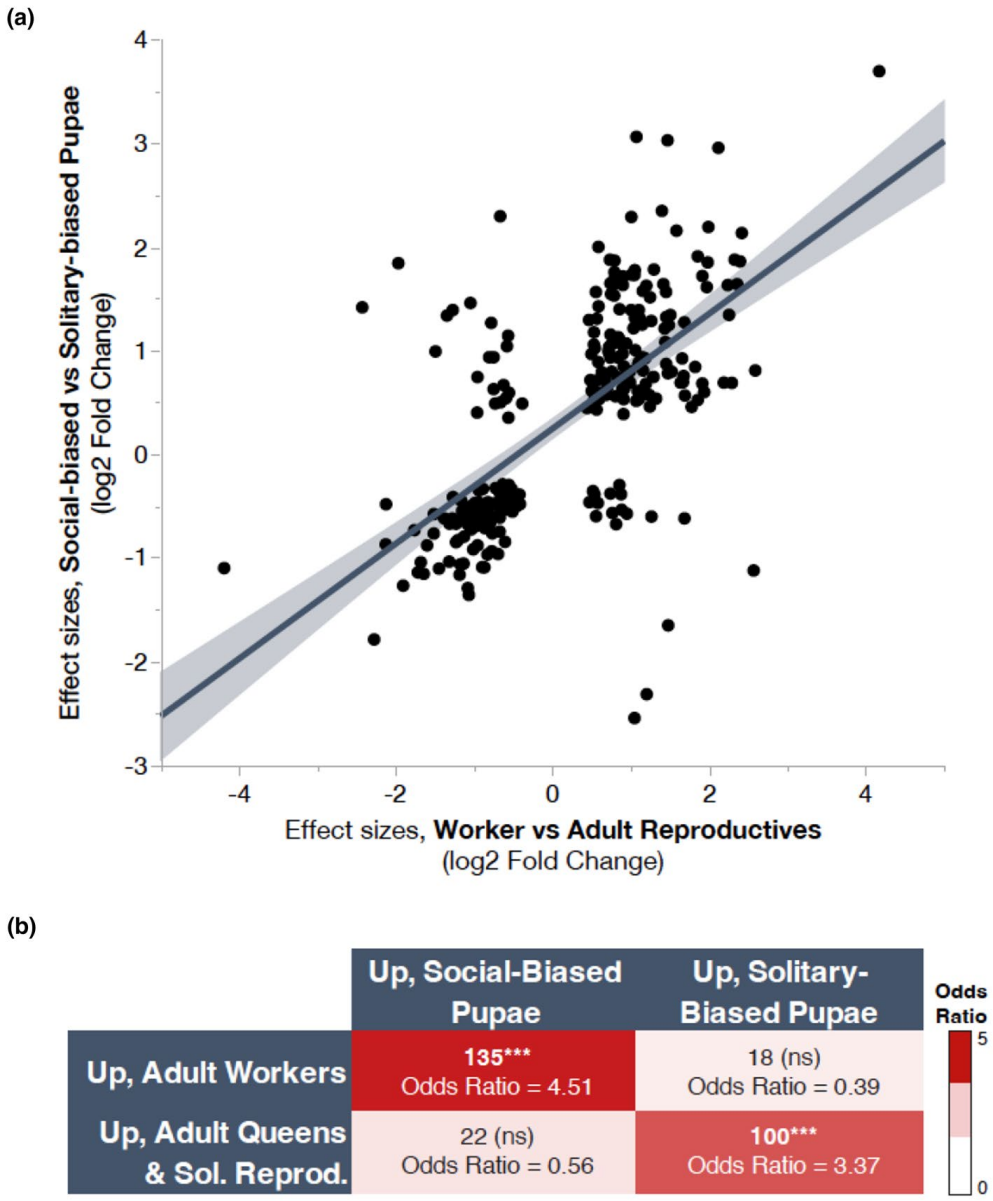
Overlapping DEGs for adult and pupal brain transcriptomes are correlated in direction and magnitude. (a) The effect size estimates (shown here as the log_2_‐fold change; DESeq2, FDR < 0.001) for overlapping brain DEGs between adult reproductives vs. workers and solitary‐biased vs. social‐biased pupae are strongly correlated (linear regression, *R* ^2^ = .41, *F* = 190.24, *p* = 3.38e‐33). Solid line represents the best fit line for the linear regression, and the shading indicates the 95% confidence intervals around this fit. (b) DEGs in adults and pupae are highly concordant in expression patterns. Genes upregulated in social‐biased pupal brains are strongly enriched for genes that are also upregulated in adult workers (hypergeometric test, *p* = 8.05e‐37, odds ratio = 4.51). Similarly, genes more highly expressed in solitary‐biased pupae are more likely to also be upregulated in adult queens and solitary reproductives (hypergeometric test, *p* = 1.58e‐20, odds ratio = 3.37). There is no significant enrichment for discordant patterns of gene expression (e.g., genes upregulated in workers do not also show an enrichment for genes upregulated in solitary‐biased pupae, etc.). Asterisks denote a significant enrichment with *p* < .0001.

Differences observed between pupae and adults are strongly concordant across social forms (Figure 4). For example, social‐biased pupae are predicted to develop into adult workers, and genes upregulated in these pupae overlap significantly with genes upregulated in adult workers. Similarly, solitary‐biased pupae are predicted to develop into reproductive adults, and we also observe a significant overlap between genes upregulated in this group and genes upregulated in queens and solitary reproductives. Moreover, the effect size estimates (measured as log_2_ fold changes from DESeq2) for adult reproductives versus workers and solitary‐biased versus social‐biased pupae are strongly correlated (Figure 4a; linear regression, *R* ^2^ = .41, *F* = 190.24, *p* = 3.38e‐33), suggesting that both the magnitude and the directionality of these expression changes are similar in pupal and adult brains. These genes show substantial GO enrichment for chemical synaptic transmission, GABAergic signalling, neurotransmitter receptor metabolism, and synaptic processes, among others (Figure S15). Finally, a random forest classifier demonstrates that pupal brain gene expression is highly predictive of adult behavioural states (Figure S16).

In the fat bodies, we identified 2028 DEGs between social‐biased and solitary‐biased pupae. GO enrichment analyses reveal that genes downregulated in solitary‐biased pupae include cell division, cell cycle, and the Toll signalling pathway (to a lesser degree). Genes upregulated in social‐biased pupae are again enriched for processes linked to cytoplasmic translation, translation, and actin filament bundle assembly, among others (Figure S17). Similar to brains, we found that 321 of these DEGs are also differentially expressed in the fat bodies of adult reproductives and workers; this is significantly more than expected by chance (hypergeometric test, *p* = 8.18e‐10, odds ratio = 1.55). Again, we observe similar concordance across pupal and adult DEGs (Figure S18). As in the brain, we see a significant correlation between effect size estimates (e.g., log2 fold change from DESeq2) across both adult and pupal comparisons (Figure S18a; linear regression, *R* ^2^ = .225, *F* = 92.36, *p* = 2.27e‐19).

## 4 DISCUSSION

Here, we explore some of the environmental and molecular factors associated with the variable social strategies employed by *L. baleicum*. In this socially flexible species, females can produce either social or solitary nests, and this variation is tightly linked to nesting locality, as supported by previous studies (Cronin & Hirata, 2003; Hirata & Higashi, 2008). Nests constructed in shady patches of the aggregation are largely solitary, while those constructed in sunnier patches are eusocial and contain a reproductive queen and 2–3 non‐reproductive workers. This variation is most likely due to behavioural plasticity – there appears to be no genetic divergence between social and solitary nests in this population (Hirata & Higashi, 2008), though more detailed genetic data are needed.

### 4.1 Spring soil temperature is a strong predictor of social behaviour

We found that soil temperatures in early spring, but not in mid‐summer, are a strong predictor of the social structure of *L. baleicum* nests. Sites with cooler spring soil temperatures are highly biased towards solitary nests, while sites with warmer spring temperatures are highly biased towards social nests. Summer soil temperatures were not associated with this variation, suggesting that females adjust their nesting strategy depending on early‐spring soil conditions at their chosen nest site.

Our results are highly consistent with natural variation in social behaviour that has been documented in many social systems (Cronin, 2001; Cronin & Schwarz, 1999b; Eickwort et al., 1996; Field et al., 2010; Kocher et al., 2014; Lawson et al., 2018; Plateaux‐Quénu, 2008; Purcell, 2011; Rubenstein & Lovette, 2007; Sakagami & Munakata, 1972). Within the social insects, social variation typically occurs across elevational (Sakagami & Munakata, 1972; Soucy & Danforth, 2002) or latitudinal (Cronin & Schwarz, 1999a; Davison & Field, 2016; Field et al., 2010) gradients, and solitary nesting strategies are most prevalent when season lengths for foraging and reproduction are shorter.

These geographical patterns in social behaviour can be explained, at least in part, by combined constraints in season length as well as the length of the colony reproductive cycle (Kocher et al., 2014). While solitary nests can immediately produce the next generation of reproductives, eusocial nests typically raise non‐reproductive workers prior to rearing the next reproductive generation (Michener, 1974; Quiñones & Pen, 2017). In environments with shorter seasons, eusocial nests may simply not have sufficient time to complete reproduction, and season length may therefore act as a strong selective force that shapes variation in social behaviour (Kocher et al., 2014; Purcell, 2011).

Our findings demonstrate that early‐spring temperatures can similarly predict social structure within a local thermal gradient. Much like variation in season length across elevational or latitudinal gradients, the differences in soil temperatures between the shady and sunny sides of the aggregation could also impact the amount of time available for bees to reproduce and for offspring to develop. The soil temperatures at sunny sites are much warmer in the spring, suggesting that nests in these parts of the aggregation can begin provisioning and egg laying sooner than their shady‐side counterparts, potentially providing them with additional time required to produce two broods rather than just one. Thus, temperature and time constraints can likely shape variation in social behaviour at both local and broader geographic scales.

### 4.2 Reproductive status is the strongest predictor of adult gene expression

To better understand the molecular underpinnings of this plasticity, we characterized variation in the gene expression profiles of adult females from solitary and social nests. We find that reproductive status is a strong predictor of gene expression – queens, foundresses, and solitary reproductive all exhibited similar patterns of brain gene expression that were distinct from that of workers. Both social queens and solitary reproductives had highly similar gene expression patterns to each other and to spring foundresses.

Workers in social nests represent the most distinct group in terms of gene expression profiles. Workers do not vary in body size compared to reproductive females, but they do vary in ovarian activation. It is possible that some of these differences could be age‐associated because workers have not undergone diapause. Our mid‐season sampling of reproductive females was likely to include both overwintered adult females and same‐season females from the first brood that either inherited nests as replacement queens or established new nests as solitary females. Thus, the gene expression differences we observed are most likely to be driven by differences in reproductive state rather than behavioural types.

Differentially expressed genes between adult queens and workers were largely associated with neural processes and reproduction. In adult fat bodies, *juvenile hormone binding protein 14* (*jhbp14*) is the most strongly upregulated gene in queens compared to workers. Juvenile hormone (JH) is predicted to be involved in various multicellular organism reproductions, and *jhbp14* is involved in the transportation of JH from the *corpora allata* (the glands where JH is synthesized) to all other target cells (Kolodziejczyk et al., 2008). In many insects, JH is a known regulator of development, gonadal activities, diapause, caste determination, reproductive behaviours (Brent et al., 2016; Giray et al., 2005; Nijhout, 1994), and a modulator of sexual receptivity (Bilen et al., 2013; Ringo, 1996; Ringo et al., 1991). Selection on the JH signalling pathway has also been associated with the evolution of social behaviour in sweat bees (Jones et al., 2023).

### 4.3 Pupal gene expression varies across shady, solitary‐biased and sunny, social‐biased sites

We identified gene expression differences in developing pupae that were associated with variation in environmental conditions among sites. Given the strong correlation between spring soil temperatures and nesting behaviour, the gene expression differences we observe are likely to be predictive of future reproductive status: pupae from cooler, solitary‐biased nests are predicted to develop into future reproductive females while pupae from warmer, social‐biased nests are predicted to develop into non‐reproductive workers.

The top DEGs in these groups are largely uncharacterized but also include genes associated with chitin‐binding activity and *Osiris 8*. Members of the *Osiris* gene family are known to contribute to phenotypic plasticity in a wide range of social insect species (Smith et al., 2018).

### 4.4 Pupal expression differences are predictive of adult social forms

We found substantial overlap between the pupal and adult DEGs, and this finding was supported by more rigorous testing using a random forest classifier to demonstrate that gene expression profiles of pupae can be used to predict adult behaviour. There is a strong enrichment for concordant expression differences between pupal and adult social forms. For example, social‐biased pupae are predicted to develop into adult workers, and genes upregulated in these pupae overlap significantly with genes upregulated in adult workers in both brain and fat body. Similarly, solitary‐biased pupae are predicted to develop into reproductive adults, and we observe a significant overlap between genes upregulated in the brains of this group and genes upregulated in queens and solitary reproductives. It seems unlikely that variation in soil temperatures alone can explain the observed overlap in expression patterns because soil temperatures were only significantly correlated with nesting behaviours in the spring when pupae were collected and not in the summer when adult queens, workers, and solitary reproductives were collected. Interestingly, the concordance between solitary‐biased pupae and reproductive adults is less pronounced in the fat bodies of these groups. This difference could potentially be associated with the higher fat stores often observed in newly eclosed adults with high reproductive potential; while developing females grow these fat stores, reproductively active queens and solitary females deplete these fat stores throughout their reproductive period. Finally, for both solitary‐ and social‐biased pupal versus adult comparisons, we find a strong correlation between the effect size estimates of the DEGs in both brains and fat bodies. Taken together, these findings suggest that the gene expression differences in pupae are predictive of adult social behaviours.

Several of the DEGs with concordant gene expression patterns have previously been linked to variation in adult social behaviour, but their expression patterns in developing pupae have rarely been characterized. For example, we find that *syntaxin‐1a* (*Syx1a*) is upregulated in both social‐biased pupae and adult workers. *Syx1a* is a member of the SNAP/SNARE complex and plays a key role in neurotransmission (Schulze et al., 1995). The expression differences we observe in *L. baleicum* mirror changes in gene expression documented between social and solitary populations of another socially variable halictine bee, *Lasioglossum albipes* (Kocher et al., 2018). Our results suggest that changes in *syx1a* expression are not only associated with variation in adult social behaviours in multiple sweat bee species, but that these expression differences may also be relevant during earlier developmental stages. In addition to *syx1a*, we also find concordant changes in gene expression between pupae and adults for a number of genes previously linked to variation in adult social behaviours, including *Tyramine receptor* (*Tyr1*), *NMDA receptor* (*Nmdar1*), *Excitatory amino acid transporter 1* (*Eaat1*), and *Fragile X messenger ribonucleoprotein 1* (*Fmr1*). Each of these genes has been linked to variation in adult social behaviour in several social insect systems (Arinrad et al., 2021; Mineur et al., 2006; Spencer et al., 2005; Ujita et al., 2018; Wang et al., 2020). More broadly, these concordant DEGs between pupal and adult brains are strongly enriched for synaptic transmission, long‐term memory, and GABAergic signalling, suggesting that at least some of the neurobiological changes linked to adult social behaviours may be established earlier during development.

### 4.5 Developmental environments can shape adult social behaviours in *L. baleicum*

In many organisms, the conditions that occur during early life‐sensitive periods can generate lasting and sometimes irreversible effects on developmental trajectories (English & Barreaux, 2020). For example, in many social insects, nutritional inequities during early larval development differentiate individuals irreversibly into reproductive queens or non‐reproductive workers. Although all adult females in *L. baleicum* are capable of switching between social forms, our results suggest there may also be key differences that take place during *L. baleicum* development that sets the stage for adult reproductive status and social behaviours.

Differences in soil temperature could potentially alter the developmental trajectories of offspring in the spring brood. For example, changes in developmental rates could alter expression of different transcriptional networks in developing brood, potentially biasing developmental outcomes towards reproductive states. Warmer temperatures are well known to accelerate development times in many insects (Cui et al., 2018; Van Dis et al., 2021). These effects have also been demonstrated directly in *L. baleicum*, where incubation of brood at different temperatures shifts the development times of pupae (Hirata & Higashi, 2008). Similarly, studies of the small carpenter bee, *Ceratina calcarata*, have also demonstrated that warmer developmental environments decrease development times but that they also lead to reduced adult body size and lower survivorship for these individuals (DeHaan et al., 2022).

An additional possibility is that females are differentially provisioning offspring to bias the development of their offspring towards reproductive states (Kapheim et al., 2011). Studies in other bee species have shown that increasing the provision sizes to pupae can alter body size and reproductive status (Lawson et al., 2017; Richards & Packer, 1996). Moreover, in *C. calcarata*, maternal provisions of less nutritious pollen and smaller provision sizes can lead to the production of smaller daughters with less reproductive potential (Lawson et al., 2016). These “dwarf eldest daughters” are more likely to remain in the nest as guards, increasing the fitness benefits to the mother. Whether or not differences in maternal provisions influence caste development in *L. baleicum* remains to be discovered.

While this study documents a strong correlation between spring soil temperature and social behaviour, it remains to be determined whether or not differences in developmental temperatures or other differences, such as developmental diets or maternal manipulation, are the causal factors. Future studies that incorporate finer‐scale developmental sampling with environmental manipulations of temperature or diet in sweat bees could provide novel insights into how distinct transcriptional patterns set the stage for distinct behavioural forms in adults.

## 5 CONCLUSIONS

Our study provides insights into how environmental factors can shape behavioural variation in a socially flexible sweat bee. Our results reveal that spring soil temperatures are highly predictive of nest social structure and are consistent with theory predicting a trade‐off between the reproductive benefits of social nesting versus adequate season lengths required for social reproduction. We find that the primary molecular correlates of adult social behaviours are associated with reproductive status; there are no major differences in the brain gene expression profiles of social versus solitary reproductive females, but workers have a distinct transcriptomic profile. Surprisingly, our results also suggest that the behavioural and reproductive plasticity in this system may not be quite as flexible as previously assumed. We find that developing pupae from social‐ and solitary‐biased nesting sites have distinct transcriptomic profiles, suggesting that the developmental environment may set the stage for adult behaviours even in socially flexible, totipotent species.

## AUTHOR CONTRIBUTIONS

KSO, SDK, and ALC designed and conceptualized the study. Samples were collected by KSO, SDK, TTHN, ALC, and SVG. KSO generated genomic data, and data curation was handled by KSO and AEW. KSO, IMT, and SDK analysed genomic data. KSO and SDK generated figures and wrote the initial draft of the manuscript. SDK supervised the work. All authors reviewed and edited the final manuscript.

## CONFLICT OF INTEREST STATEMENT

Authors declare that research was conducted in the absence of any commercial or financial relationships that could be construed as a potential conflict of interest.

## Supporting information


Appendix S1.

## Data Availability

Research collaborations were established with scientists from the countries providing genetic samples, and all collaborators are included as coauthors. Our data and results are also available on public databases, extending benefits to the broader scientific community. The *L. baleicum* genome assembly is available on NCBI, accession GCA_022376115.1. Raw sequence data for all samples have been deposited in NCBI, SRA submission, SUB12095212.
